# *In vitro *antioxidant activity of alkaloids from southern Brazilian *Psychotria*: a comparative analysis

**DOI:** 10.1186/1753-6561-8-S4-P237

**Published:** 2014-10-01

**Authors:** Mariana Rau, Hélio Matsuura, Arthur Germano Fett-Neto

**Affiliations:** 1 Universidade Federal do Rio Grande do Sul, Porto Alegre, RS, Brazil

## Background

Monoterpene índole alkaloids (MIAs) comprise a class of secondary metabolites of dual biosynthetic origin, characterized by the presence of an indole and a terpene portion. MIAs are widely studied because of their bioactive properties and pharmacological potential. In this study, major MIAs from native species of *Psychotria *of southern Brazil were used: *P. brachyceras, P. umbellata *and *P. leiocarpa *share the same habitat and accumulate the alkaloids brachycerine, psychollatine and N,β-D-glucopyranosyl vincosamide, respectively. The three MIAs are related and have structural similarities. Previous work identified antioxidant properties of these alkaloids and induction of their accumulation by oxidative stress, suggesting that they play roles as antioxidant compounds, something regarded as unusual for this metabolite class [1]. MIAs may have a role on control of oxidative burst generated by stress, aiming at reducing the reactive oxygen species' harmful effects and promoting the maintenance of plant fitness. Herein, the antioxidant potential of the three alkaloids against superoxide anions and singlet oxygen is comparatively evaluated, and putative structure-activity relations are considered.

## Methods

The antioxidant capacities were evaluated according to previously established protocols. Quenching activity against superoxide anions was measured by Nitro Blue Tetrazolium (NBT) reduction (solution coloration turns blue) by spectrophotometry at 560nm [2]. The singlet oxygen quenching was assessed by rubrene color decay monitored by spectrophotometry at 440nm [3]. The alkaloids were tested in equimolar concentrations (5mM for superoxide and 2mM for singlet oxygen). The positive control was Trolox™ (vitamin E synthetic analogue) and the negative control was performed by addition of the solvent solution, only. A comparison was also done with the flavonoid rutin, a well known natural antioxidant phenolic, at the same concentration. Antioxidant capacity is presented in percentage of reduced NBT, in IC50 of antioxidant activity (alkaloid concentration able to mitigate 50% of ROS) and by rubrene color decay.

## Results

Superoxide anions: The alkaloids GPV and brachycerine decreased NBT reduction rate to under 10%, result comparable to the positive control (Trolox™), which decreased to 15%. Psychollatine brought reduction down to 27%. The reaction without the quenchers achieved 90% of NBT reduction. The alkaloids quencher activity of superoxide was comparable to that of the flavonoid rutin. IC50 activity against superoxide yielded the following results: GPV was able to mitigate 50% of ROS at the lowest concentration, followed by brachycerine and psychollatine (0.0475mM, 0.0624mM and 0.0637mM, respectively). These values were higher than but comparable to those of rutin (0.0224mM). Singlet oxygen: quenching activity was similar for GPV, psychollatine and Trolox™, observable by the lower decay of rubrene color. Brachycerine was not so efficient, but still better than the negative control.

## Conclusions

Structural groups such as rings, double bonds, amines and glycosylations affect the efficiency of antioxidant activity. GPV appeared to have the best antioxidant activity in both assays, probably as a result of the contribution of a tertiary amine and additional glycosylation for combating ROS. Despite the significant structure similarity between brachycerine and psychollatine, these metabolites did exhibit specificity in ROS mitigation: the first was more efficient against superoxide anions and the second, against singlet oxygen.
